# Osteoprotegerin and RANKL-RANK-OPG-TRAIL signalling axis in heart failure and other cardiovascular diseases

**DOI:** 10.1007/s10741-021-10153-2

**Published:** 2021-07-27

**Authors:** Mieczysław Dutka, Rafał Bobiński, Wojciech Wojakowski, Tomasz Francuz, Celina Pająk, Karolina Zimmer

**Affiliations:** 1 Department of Biochemistry and Molecular Biology, Faculty of Health Sciences, University of Bielsko-Biala, Willowa St. 2, 43-309 Bielsko-Biała, Poland; 2 Department of Cardiology and Structural Heart Disease, Medical University of Silesia, Katowice, Poland; 3 Department of Biochemistry, Medical University of Silesia, Katowice, Poland

**Keywords:** Osteoprotegerin, TRAIL, RANK, Myocardial infarction, Heart failure, Endothelial cells

## Abstract

Osteoprotegerin (OPG) is a glycoprotein involved in the regulation of bone remodelling. OPG regulates osteoclast activity by blocking the interaction between the receptor activator of nuclear factor kappa B (RANK) and its ligand (RANKL). More and more studies confirm the relationship between OPG and cardiovascular diseases. Numerous studies have confirmed that a high plasma concentration of OPG and a low concentration of tumour necrosis factor–related apoptosis inducing ligand (TRAIL) together with a high OPG/TRAIL ratio are predictors of poor prognosis in patients with myocardial infarction. A high plasma OPG concentration and a high ratio of OPG/TRAIL in the acute myocardial infarction are a prognostic indicator of adverse left ventricular remodelling and of the development of heart failure. Ever more data indicates the participation of OPG in the regulation of the function of vascular endothelial cells and the initiation of the atherosclerotic process in the arteries. Additionally, it has been shown that TRAIL has a protective effect on blood vessels and exerts an anti-atherosclerotic effect. The mechanisms of action of both OPG and TRAIL within the cells of the vascular wall are complex and remain largely unclear. However, these mechanisms of action as well as their interaction in the local vascular environment are of great interest to researchers. This article presents the current state of knowledge on the mechanisms of action of OPG and TRAIL in the circulatory system and their role in cardiovascular diseases. Understanding these mechanisms may allow their use as a therapeutic target in cardiovascular diseases in the future.

## Introduction

Osteoprotegerin (OPG) is a glycoprotein that belongs to the tumour necrosis factor superfamily (TNFSF). TNFSF is a large group of structurally and functionally related cytokines, currently comprising more than 20 different ligands and more than 30 associated receptors, all of which make up the tumour necrosis factor receptor superfamily (TNFRSF) [1]. OPG belongs to this group of receptors and is identified by the symbol OPG/TNFRSF11B. It belongs to the group of soluble receptors and occurs mainly as a free form, not bound to a cell membrane [1–3].

OPG consists of 401 amino acids with a molecular weight of 60 kD. There are 7 structural domains in the OPG molecule, which determine its specific biological activities. OPG is secreted as a monomer (60 kD) and as a disulphide-linked homodimer (120 kD). In the circulating blood, OPG occurs in the form of a monomer, a homodimer, and also as OPG combined with its ligands — receptor activator of nuclear factor kappa B ligand (RANKL) and TNF-related apoptosis inducing ligand (TRAIL) [3–5].

OPG is known mainly as a regulator of bone remodelling under physiological conditions and in various clinical settings. The effect of OPG on bone metabolism is to block the interaction between the receptor activator of nuclear factor kappa B (RANK) and its ligand RANKL, and this is achieved by its ‘decoy receptor’ function [3–5]. RANKL itself is a major activator of osteoclast differentiation and maturation, thus promoting osteoclastogenesis. OPG, by blocking the connection between RANKL and its receptor RANK, inhibits osteoclast maturation and osteoclastogenesis, and thus inhibits the osteolysis processes [5, 6]. OPG is produced in various organs such as the kidneys, intestines, stomach and bones, as well as in the cells of the extracellular matrix, megakaryocytes, cells of the immune system such as B lymphocytes or dendritic cells and, very importantly, vascular endothelial cells (ECs) and vascular smooth muscle cells (VSMCs) [5, 6]. It has also been confirmed that granulocytes have the ability to produce OPG and interleukin 17 (IL-17). IL-17 increases the recruitment of granulocytes when inflammation occurs and induces the production of various pro-inflammatory factors [4]. Activated T lymphocytes also induce OPG expression in osteoblasts through interleukin 4 (IL-4) and interleukin 13 (IL-13). The participation of OPG in the regulation of the activity of dendritic cells and the function of T and B lymphocytes has also been shown [1, 6–9].

There is more and more research data confirming that, apart from its role in regulating bone metabolism through the OPG/RANKL/RANK axis, OPG may also participate in cardiovascular disease (CVD) processes or be a prognostic indicator of CVDs [7]. The calcification observed in atherosclerotic plaques is a factor in the pathogenesis of atherosclerosis and is associated with the morbidity caused by atherosclerosis. This calcification is subject to regulatory mechanisms similar to those in bone tissue, hence the initial interest in OPG and the OPG/RANKL/RANK axis in the context of CVDs, especially when the first studies of OPG knockout mice showed that, apart from severe osteoporosis, there was also increased calcification of the aortic wall. However, later clinical observations showed the opposite results [10]. It was found that high concentrations of OPG are associated with a higher cardiovascular (CV) risk in patients with coronary artery disease (CAD) [3, 5, 6, 10–12]. It has been shown that in diabetic patients, higher levels of OPG are associated with both a greater severity of atherosclerotic lesions and a higher degree of calcification in the coronary arteries [13]. Higher concentrations of OPG were also found in patients with retinopathy and diabetic neuropathy and in patients with arterial hypertension [7]. Therefore, an increased concentration of OPG is regarded as a marker of vascular pathologies associated with diabetes and arterial hypertension, as well as an indicator of endothelial dysfunction and high CV risk [7, 10]. In patients with type 2 diabetes, confirmation that high plasma concentrations of OPG are associated with the presence of increased endothelial dysfunction was established by assessing the endothelial function in these patients in vivo using flow-mediated dilation (FMD) measurement of the brachial artery [12]. Another group of researchers evaluated patients with newly diagnosed type 2 diabetes who had significantly higher plasma concentrations of OPG compared to a control group without diabetes [14]. In this group of patients, it was confirmed that the initiation of the pharmacological treatment for the diabetes caused a significant decrease in OPG plasma concentration. This, however, was still higher than in the control group without diabetes. The study confirmed, moreover, that the impaired endothelial function, initially found in the group of diabetic patients and assessed in vivo by brachial artery FMD, improved 6 months after the initiation of diabetes treatment. However, the results of FMD were still worse than in the group of healthy people [14]. It was also confirmed in a mouse model of dyslipidaemia and diabetes that the occurrence of diabetes is associated with a higher level of OPG expression in the aortic wall when compared to non-diabetic mice [15]. In addition, OPG plasma concentrations were significantly higher in mice with induced diabetes. It was also confirmed in this study that the plasma concentration of cholesterol and glycaemia correlates not only with the plasma concentration of OPG but also with the severity of the atherosclerotic process in these mice [15]. The mechanisms of action of OPG that could explain its pathogenetic role in damage to the arterial wall and development of atherosclerotic plaque remain unclear. It is believed that OPG contributes, among other things, to the development of inflammation within the vascular wall and escalates the adhesion of leucocytes to the vascular endothelium [16, 17]. These changes, observed in the early stages of the development of endothelial dysfunction, trigger the development of atherosclerotic vascular damage. In addition, there is an increase in the significance of angiotensin II (Ang II), platelet-derived growth factor (PDGF) and basic fibroblast growth factor (bFGF), which can stimulate OPG expression in VSMCs and contribute to the development of atherosclerotic lesions [7].

## OPG in cardiovascular diseases: clinical observations

Significantly higher OPG plasma concentration has been confirmed in patients with CAD compared to healthy volunteers, and in a group of patients diagnosed with CAD, these high concentrations of OPG are associated with a greater range of atherosclerotic lesions in the coronary arteries and a higher risk of death [3, 5, 6, 10–12, 18]. It has been confirmed that high concentrations of OPG are a predictor of a higher frequency of hospitalisations due to exacerbated ischaemic heart failure (HF) with a reduced ejection fraction [5]. Significant changes in OPG plasma concentrations were also observed in patients with unstable angina (UA) and in patients with acute myocardial infarction (AMI) [11, 19–22]. The increased concentration of OPG in CAD, in the course of acute coronary syndromes, is considered an indicator of the risk of adverse CV events and poor prognosis [11, 20, 21]. In studies evaluating a series of patients admitted to hospital with ST-segment elevation myocardial infarction (STEMI) and given percutaneous coronary intervention (PCI), high plasma concentrations of OPG turned out to be an independent predictive factor of death, recurrent myocardial infarction (MI) and hospitalisation for HF, even after taking into account the classic factors of poor prognosis [11, 21]. Another study confirmed that in patients with STEMI, high plasma concentrations of OPG are associated with a statistically significant increase in the risk of major adverse cardiac and cerebrovascular events (MACCEs), despite the lack of such an association between OPG plasma concentration and the size of the necrotic area [20]. In patients with STEMI treated with primary coronary angioplasty, a relationship has also been demonstrated between a high plasma OPG concentration on admission to the hospital and the frequency of no-reflow phenomenon and the development of adverse post-infarction left ventricular remodelling [22]. There was a significant increase in the plasma concentration of OPG in both UA and AMI patients compared to healthy individuals [19]. On the other hand, another parameter, often assessed in conjunction with OPG, namely TRAIL, did not show significant changes in concentrations in UA patients compared to healthy controls. However, in patients with AMI, significantly lower levels of TRAIL were found, which, combined with an increase in the concentration of OPG, resulted in a significant increase in the OPG/TRAIL ratio in this group of patients. Due to the increase in OPG concentration in patients with UA, even though there was no decrease in TRAIL concentration, a significant increase in the OPG/TRAIL ratio was found in patients with UA. However, it should be emphasised that the value of the OPG/TRAIL ratio was significantly higher in patients with AMI than with UA [19]. The OPG/TRAIL ratio is normalised in patients after MI after about 6 months [19].

**Fig. 1:**
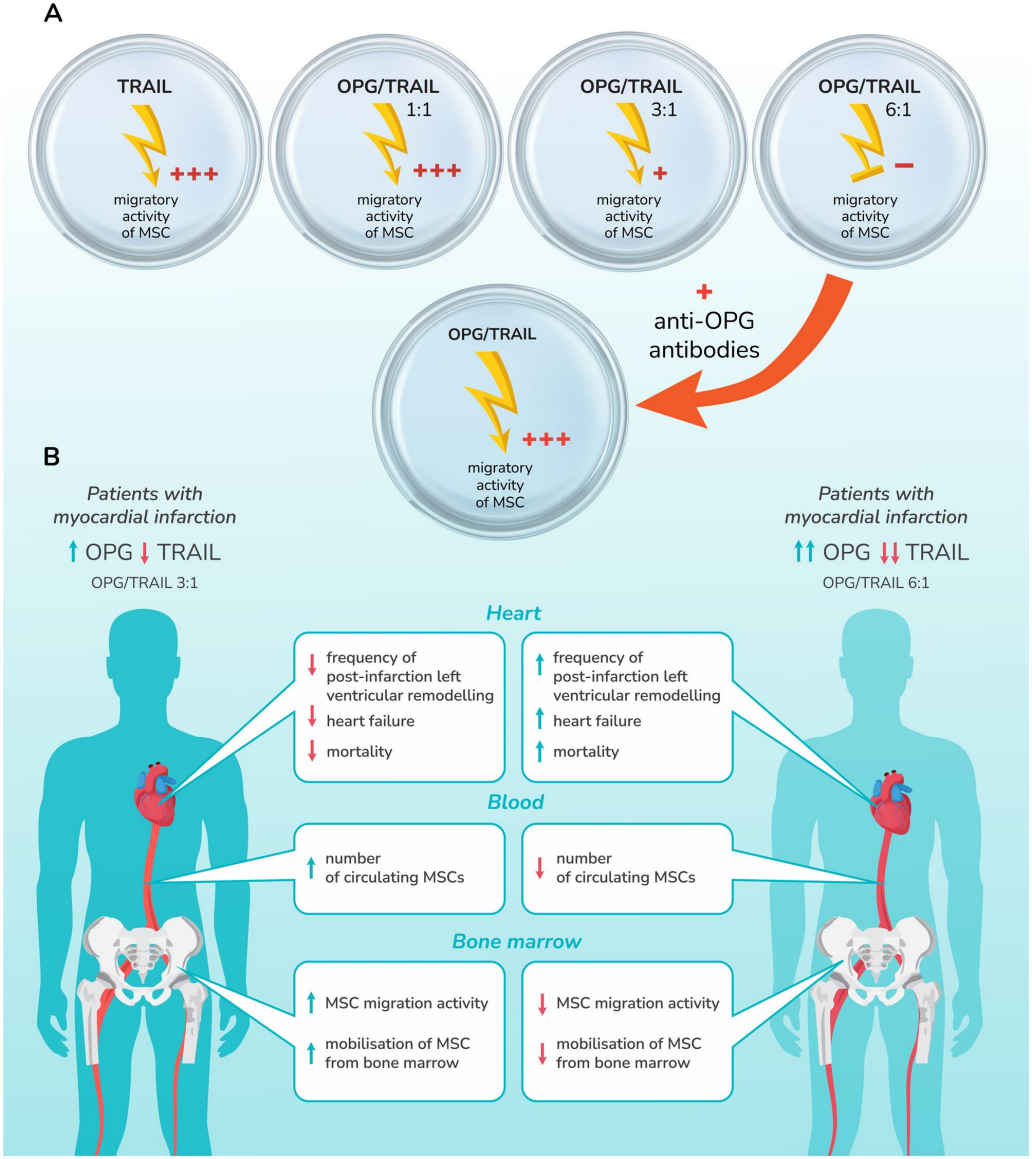
The impact of TRAIL and OPG on the migrating ability of MSCs. (A) In vitro studies. TRAIL strongly increases the migratory activity of MSCs. The addition of OPG results in inhibition at an OPG/TRAIL ratio of 3:1, and in the case of a higher concentration of OPG (OPG/TRAIL 6:1) in the abolition of the stimulating effect of TRAIL on the migrating ability of MSCs. The addition of anti-OPG antibodies to this system restores the stimulating effect of TRAIL on the migrating ability of MSCs. (B) In vivo studies. It was found in clinical studies that patients who develop HF after MI have significantly higher OPG concentrations and a higher OPG/TRAIL ratio compared to patients who do not develop HF after MI. Clinical trials confirm that patients with MI and a higher OPG/TRAIL ratio (6:1) are more likely to develop post-infarction left ventricular remodelling, develop HF more frequently and have a higher mortality compared to those with MI and a lower OPG/TRAIL ratio (3:1). Moreover, in this group of patients with MI and a high OPG/TRAIL ratio (6:1), a lower mobilisation of MSC from bone marrow and a lower number of circulating MSCs in the peripheral blood were confirmed compared to patients with MI with a lower OPG/TRAIL ratio (3: 1). Explanation of abbreviations in the main text

Many different studies confirm the occurrence of significant changes in the plasma concentrations of cytokines and soluble receptors of the TNFRSF in patients in the acute phase of MI [1, 19–21, 23, 24]. These changes include tumour necrosis factor-alpha (TNF-alpha, OPG and TRAIL, among others. These studies confirmed, in particular, a significant increase in the plasma concentration of TNF-alpha and OPG, and a decrease in the concentration of TRAIL and, consequently, an increase in the ratio of OPG/TRAIL in the plasma of patients in the acute phase of MI compared to healthy individuals [19, 23]. A high plasma concentration of OPG, and particularly a high OPG/TRAIL ratio, was also confirmed as a strong predictor of CV mortality in patients with both STEMI and non ST-segment elevation myocardial infarction (NSTEMI). High concentrations of OPG and a high OPG/TRAIL ratio were associated with significantly higher early (30-day) and late (1-year) mortality in this group of patients [21, 24]. It has been shown that the higher post-MI mortality in patients with a high concentration of OPG and a high OPG/TRAIL ratio was mainly related to adverse post-infarction left ventricular remodelling and the development of HF. No such relationship, however, has been confirmed in terms of electrical instability and recurrent myocardial ischaemia [24]. Therefore, attempts are being made to understand the mechanisms determining the adverse remodelling of the left ventricle and the development of HF in some patients after MI, as well as the protective mechanisms that prevent such remodelling in other patients. Among the factors related to these mechanisms, in addition to the above-mentioned cytokines and members of the TNFRSF, there is a growing interest in myeloid stem and progenitor cells, including mesenchymal stem cells (MSCs). These are mobilised from the bone marrow when organ damage occurs, as in AMI [23, 25, 26]. The degree of this mobilisation and, consequently, the number of MSCs circulating in the peripheral blood, which can potentially be involved in the repair processes, vary. The mechanisms for this variation are not fully understood. It is known from follow-up observations, however, that the number of MSCs in peripheral blood in the acute phase of MI is significantly lower in patients with adverse remodelling of the left ventricle and the development of HF, compared to those who do not develop adverse left ventricle remodelling and HF after MI [23]. Moreover, it was found that patients who develop HF after MI have significantly higher OPG concentrations and a higher OPG/TRAIL ratio compared to patients who do not develop HF after MI (Fig. 1). The role of TNF-alpha, OPG and TRAIL in regulating the migration capacity and mobilisation of MSCs from bone marrow has been studied in vitro [23, 27]. These studies confirmed that TRAIL has a strong stimulating effect on MSC migration, and this effect is additionally enhanced by TNF-alpha [27]. In contrast, the addition of OPG to this system under cell culture conditions strongly and dose-dependently inhibited the MSC migratory response to TRAIL. Human levels of OPG and TRAIL were used in this study. The OPG/TRAIL ratio turned out to be the key. If the OPG/TRAIL ratio was 1:1, i.e. the same as in healthy people, then OPG did not inhibit the MSC migration response in relation to TRAIL. If the OPG/TRAIL ratio was 3:1, the migratory capacity of the MSC was strongly inhibited. When the OPG/TRAIL ratio was 6:1, however, the MSC migration response to TRAIL completely disappeared [23]. In the same study, it was shown that the addition of anti-OPG antibodies, blocking the ability of OPG to bind to TRAIL, completely restored the stimulating effect of TRAIL on the migratory capacity of MSCs [23] (Fig. 1). This seems particularly interesting in the context of attempts to understand the protective mechanisms that prevent adverse left ventricular remodelling and HF after MI. In all patients with AMI, both the concentration of OPG and the OPG/TRAIL ratio increase [19]. However, in the group of patients who develop HF after MI, this increase is significantly higher (OPG/TRAIL 6:1) compared to patients who do not develop HF after MI (OPG/TRAIL 3:1) [23] (Fig. 1). It is also interesting in this context that in the above-mentioned studies, the patients who had been using statins before the MI had a significantly lower increase in TNF-alpha and OPG levels compared to patients who experienced MI without prior use of statins [23]. Other studies have confirmed that statins inhibit TNF-alpha-induced OPG release from vascular ECs [28].

In patients with chronic kidney disease (CKD), OPG also turned out to be a marker of general and CV mortality [29, 30]. Significantly higher plasma concentrations of OPG were found in a group of dialysed patients with CKD compared to both kidney transplant patients and a healthy control group [31]. In the group of patients with CKD, high levels of OPG were associated with higher all-cause mortality and CV mortality, regardless of other risk factors such as diabetes, age or the presence of CVD [29]. In addition, the OPG/TRAIL ratio showed a positive correlation with all-cause mortality and CV mortality in this group of patients, which was independent of the presence of the classic CV risk factors, with the exception of age [30]. It was also confirmed that in the group of patients with CKD, the OPG/TRAIL ratio shows a positive correlation with aortic pulse wave velocity (AoPWV) and is therefore considered a marker of vascular dysfunction in this group of patients [30].

## Potential OPG mechanisms of action in the cardiovascular system

An ever-increasing number of researchers emphasise that OPG may not only be a marker of an unfavourable prognosis in CVDs, but may also play an important pathogenetic role in their development. It has been confirmed that OPG is present in vascular ECs and in VSMCs under basic conditions, and that its production increases significantly in these cells under the influence of various stimulating factors, such as cytokines or hormones [4, 6, 17, 32]. In human aorta cells, it was confirmed that the production of OPG is 20–30 times higher in VSMCs than in ECs [6]. TNF-alpha and interleukin-1beta (IL-1B) are strong stimulators of OPG production in ECs and VSMCs. These cytokines can increase OPG expression 5–40-fold [4, 6, 32]. In ECs, OPG occurs in secretory granules, called Weibel-Palade bodies, where OPG is associated with the von Willebrand factor [4, 6].

OPG can exert its biological effect on the CV system in three ways. The first way is that it binds through a specific domain to its ligand RANKL, thereby blocking it from binding to its receptor, RANK. The second mode of action of OPG is to act directly on cells via a heparin-binding domain that has the ability to bind to heparan sulphate proteoglycans present on the surface of cells, triggering cell-surface signalling. Such OPG action has been confirmed in the area of vascular wall cells, bones and cells of the immune system [3, 6, 17, 33]. A third way in which OPG works is when it attaches to its other ligand, TRAIL. This blocks the possibility of TRAIL binding to its receptors, which weakens or abolishes the effects of TRAIL [6].

### The RANK-RANKL-OPG signalling axis and the direct action of OPG on cells

More and more is becoming known about the elements of the RANK-RANKL-OPG axis and the role of the transcription factor and nuclear factor kappa-B (NF-kB) within the ECs and their role in the pathogenesis of inflammation within the vascular wall and atherogenesis [4].

**Fig. 2:**
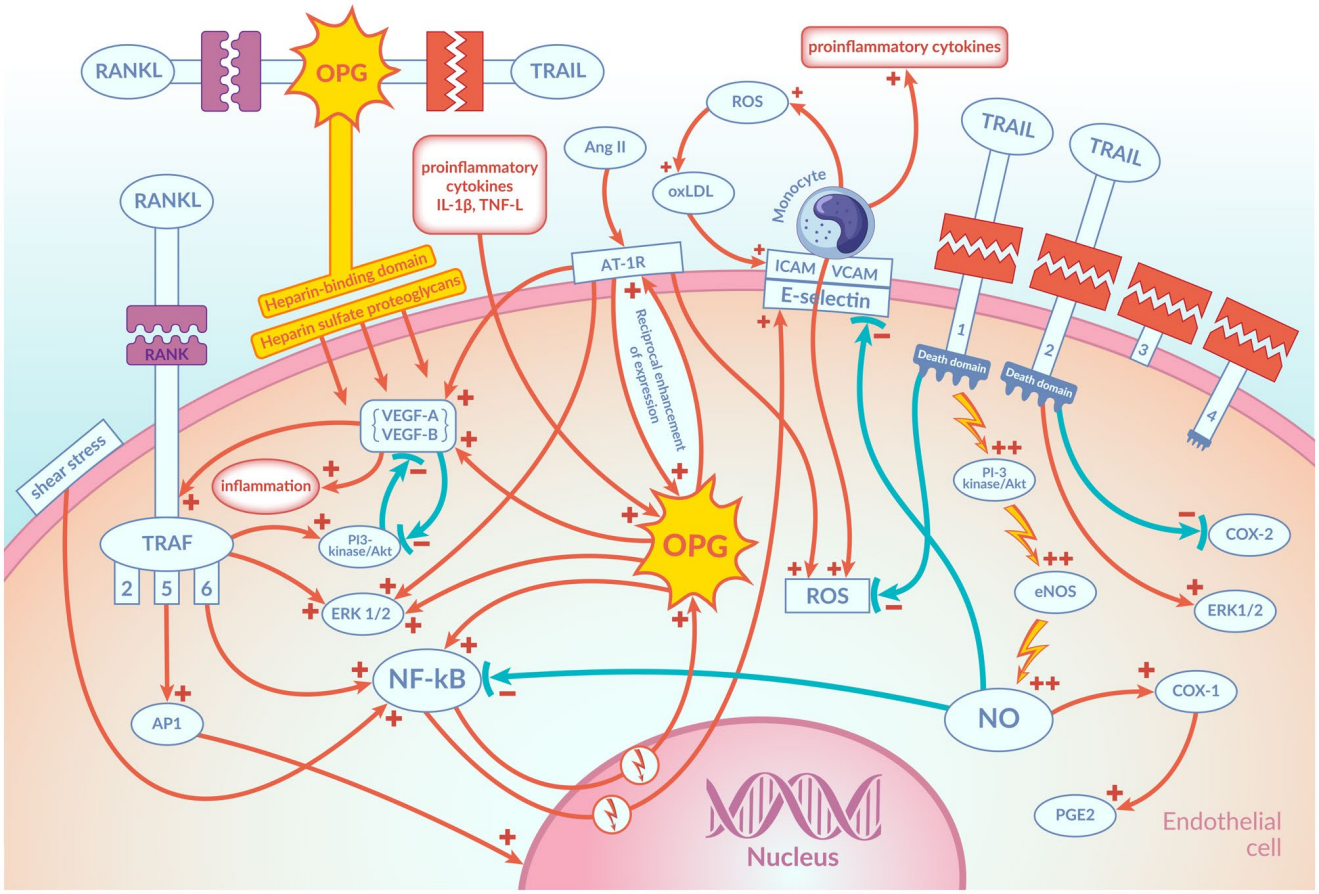
Mechanisms of action of OPG and TRAIL in endothelial cells. OPG exerts its biological effect on the endothelial cells in three ways. Firstly, it binds through a specific domain to its ligand RANKL, preventing it from binding to its receptor, RANK. Secondly, OPG acts directly on ECs via a heparin-binding domain that has the ability to bind to heparan sulphate proteoglycans present on the surface of cells, triggering cell-surface signalling. Thirdly, it attaches to its other ligand, TRAIL, preventing TRAIL from binding to its receptors, thereby weakening or abolishing its effects. The binding of RANK to its ligand RANKL results in the activation of a signalling cascade activating NF-kB and AP-1. Acting at the level of the cell nucleus, these molecules increase the expression of OPG and ICAM-1 and VCAM-1. OPG also activates NF-kB in ECs inducing the expression of ICAM-1 and VCAM-1 at the level of the cell nucleus, which leads to increased adhesion of leucocytes to the endothelial surface in the early stages of endothelial dysfunction. OPG greatly enhances the adherence of leucocytes to the surface of ECs through its heparin-binding domain. An important connection also exists between OPG and RAS. Activation of the AT1 receptors for Ang II causes, inter alia, an increase in the expression of VEGFS. VEGF-A and VEGF-B increase inflammation and remodelling in blood vessels by activating pro-inflammatory mechanisms and pathological angiogenesis, which are strengthened by OPG. Mutual stimulating interactions between OPG and RAS have also been confirmed. In ECs Ang II increases OPG expression and OPG increases the expression of the AT1 receptor for Ang II. TRAIL significantly increases the activity of eNOS and increases NO production in ECs. The augmented phosphorylation of eNOS and the increase in NO production in ECs under the influence of TRAIL occur through the activation of the PI3 kinase/Akt pathway. Additionally, TRAIL induces PGE2 production in ECs, mainly by increasing COX-1 activity. This effect of TRAIL is accomplished by increasing NO release. NO inhibits the activity of NF-kB and decreases the expression of ICAM-1, VCAM-1 and E-selectin in ECs and prevents increased adherence of leucocytes to the endothelial surface. Explanation of abbreviations in the main text

Human RANK is a protein consisting of 616 amino acids. Each of these molecules consists of the C-terminal cytoplasmic domain of 383 amino acids, a signal peptide of 28 amino acids, a transmembrane domain of 21 amino acids and an N-terminal extracellular domain of 184 amino acids [4]. RANK is a transmembrane receptor whose cytoplasmic domain is capable of binding to TNF receptor–associated factors (TRAFs 2,5,6). The binding of RANK to its ligand RANKL, which exists both as a transmembrane protein and in a soluble form (RANKLs) circulating in the blood, results in the activation of a signalling cascade activating the transcription factor NF-kB. RANKL binds to RANK as a homotrimer. The first effect of binding RANK to RANKL is the attachment of TRAFs: 2,5,6 to specific sites within the cytoplasmic domain of RANK. This triggers several signalling pathways activated by RANK/TRAF-mediated protein kinase signalling, such as inhibitory NF-kB kinase (IKK)/NF-kB and activator protein-1 (AP-1). NF-kB and AP-1 acting at the level of the cell nucleus increase the expression of OPG and adhesion molecules such as intercellular adhesion molecule 1 (ICAM-1) and vascular adhesion molecule 1 (VCAM-1), among others. It has been confirmed that OPG also activates NF-kB in ECs and thus induces the expression of ICAM-1 and VCAM-1 at the level of the cell nucleus, which leads to increased adhesion of leucocytes to the endothelial surface in the early stages of endothelial dysfunction [4, 5, 17] (Fig. 2). OPG has been shown to greatly enhance the adherence of leucocytes to the surface of endothelial cells through its heparin-binding domain. OPG binds via this domain to the sulphate proteoglycans present on the surface of an endothelial cell. This connection activates signalling pathways inside the cell, leading, inter alia, to an escalation of the adhesion of leucocytes to the EC surface [17]. In ECs, the sensors and effectors of shear stress also regulate the expression of genes regulated by NF-kB, such as the genes for ICAM-1 and VCAM-1 [4, 34]. Increased shear stress activates NF-kB, which activates gene expression for said particles at the level of the cell nucleus. However, so far, there is no confirmation that intensified shear stress increases the expression of OPG in vascular ECs. It has been confirmed, nevertheless, that shear stress increases the expression of OPG in osteocytes and reduces the influence of interleukin 17A (IL-17A) on the expression of TNF-alpha and RANKL, thus weakening the differentiation of osteoclasts [4].

In addition, low-density lipoproteins (LDLs) accumulating under the endothelial layer, and especially their oxidised forms (OxLDLs), which are involved in the induction of the atherosclerotic process at an early stage, have the ability to stimulate the expression of ICAM-1 and VCAM-1. This contributes to an escalation in the adherence of leucocytes to the endothelial surface. The degree of LDL oxidation is highly dependent on the reactive oxygen species (ROS) generated by the monocytes. The opposite effect is displayed by nitric oxide (NO), which is generated in ECs with the involvement of endothelial nitric oxide synthase (eNOS) and reduces endothelial expression of ICAM-1 and VCAM-1 [4] (Fig. 2).

The RANK-RANKL-OPG signalling axis is considered to be the main regulator of the progression of calcification of the blood vessel wall. In this process, the osteogenic differentiation of VSMCs is of fundamental importance [4]. Fibroblast growth factor 21 (FGF21) fulfils a protective role for the vessels by limiting the osteogenic differentiation of VSMCs. Ecto-5′-nucleotidase (CD73) also plays a protective role for blood vessels, by hydrolysing extracellular adenosine monophosphate (AMP) and, thus, releasing adenosine. Adenosine inhibits the calcification of the blood vessel wall [4]. Adenosine is considered to be a cytoprotective modulator that plays a protective role against tissue damage in various organs. It carries out its biological action by connecting with the G-protein-coupled receptor present on the cell surface, or rather with its subtypes A1, A2a, A2b and A3. There is a link between adenosine triphosphate (ATP)/adenosine metabolism and the RANK-RANKL-OPG signalling axis. It has been confirmed in human osteoprogenitor cells that adenosine inhibits the secretion of OPG [4].

An important connection also exists between OPG and the renin-angiotensin system (RAS), which plays a key role in the development, with age, of unfavourable changes in the VSMC and EC phenotypes and in the pathogenesis of atherosclerosis [4]. The main mediator of RAS, i.e. Ang II, acts directly on the vascular endothelium, leading to its dysfunction, the development of inflammation and the progression of atherosclerosis. Activation of the angiotensin II type 1 (AT1) receptors for Ang II causes, inter alia, an increase in the expression of vascular endothelial growth factors (VEGFs). VEGF-A and VEGF-B increase inflammation and remodelling in blood vessels by activating pro-inflammatory mechanisms and pathological angiogenesis. OPG has been confirmed to strengthen these actions of VEGFs [35]. Atheroma-derived cells harvested during endarterectomy were tested under cell culture conditions. These cells and ECs derived from human dermal microvasculature were grown with and without irbesartan, the blocker of the AT1 receptor for Ang II [36]. It has been confirmed that irbesartan, which blocks the AT1 receptor for Ang II, reduces the concentrations of interleukin-6 (IL-6), interleukin-8 (IL-8) and OPG in both cell types. Moreover, it has been shown that in the cells studied, the blockade of the AT1 receptor for Ang II leads to a reduction in extracellular signal–regulated kinase-1 and kinase-2 (ERK1 and ERK2) expression and restricts their phosphorylation. This phosphorylation of ERK1 and ERK2 is normally induced by RANKL when it is linked to RANK. Similar data was obtained in mice when another blocker of the AT1 receptor for Ang II, losartan, also inhibited RANKL-induced ERK1 and ERK2 phosphorylation [37]. This suggests a convergent action of RANKL and Ang II at the level of ERK1/2 phosphorylation regulation. OPG also has the ability to directly activate ERK1/2 phosphorylation, which is associated with pathological angiogenesis [4, 36, 37].

Moreover, mutual stimulating interactions between OPG and RAS have been confirmed [3, 38–40]. In the cells of the vascular walls of both humans and mice, Ang II was confirmed to increase OPG expression, whereas the blockade of the AT1 receptor for Ang II with Irbesartan reduces the expression of OPG in these cells. It has also been shown that, just as Ang II has a dose-dependent effect in increasing OPG expression in vascular wall cells, OPG has a dose-dependent effect in increasing the expression of the AT1 receptor for Ang II [3, 41] (Fig. 2). This is another mechanism by which OPG can promote endothelial dysfunction and the development of atherosclerotic lesions.

Under the influence of Ang II, the expression of the aforementioned VEGF is increased, and this causes overexpression of RANK in ECs and enhances the angiogenic response of these cells to RANKL. RANKL may also have an additional action in maintaining EC integrity and inducing a pro-survival effect on ECs [4]. This action of RANKL on ECs occurs via the PI3-kinase/Akt signalling pathway. OPG inhibits this effect by binding to RANKL, which prevents it from combining with RANK and activating the PI3-kinase/Akt signalling pathway. Blocking PI3-kinase reverses this pro-survival effect of RANKL in relation to ECs [42]. The PI3-kinase/Akt signalling pathway is strongly inhibited by VEGF, whose expression increases under the influence of Ang II [43] (Fig. 2).

More and more experimental data indicates the participation of OPG in promoting the inflammatory process in the blood vessel wall. OPG directly induces the expression of adhesion molecules such as ICAM-1 and VCAM-1 and E-selectin in ECs [4, 5]. Thus, OPG causes increased adherence of leucocytes to the endothelial surface, induces the early stages of endothelial dysfunction and leads to the development of atherosclerotic lesions. At the stage when enhanced recruitment of leucocytes, mainly monocytes, lymphocytes and neutrophils, from the blood takes place and they penetrate deep into the vascular wall, OPG interacts with many pro-inflammatory modulators, such as IL-6, IL-1beta or heparan sulphate proteoglycans (HSPGs) [44]. Under the influence of pro-inflammatory cytokines, the production of OPG in ECs increases significantly, which additionally enhances the expression of adhesion molecules as well as the recruitment and transmigration of monocytes, lymphocytes and neutrophils deep into the vascular wall [4, 5, 17, 45]. Many researchers emphasise the importance of OPG as a chemotactic factor for inflammatory cells infiltrating the vascular wall at an early stage of development of atherosclerotic lesions [4, 17, 44–48]. The participation of pro-inflammatory cytokines such as IL-6 and IL-1beta in this process has become the basis for the development of new therapeutic strategies. Attempts are being made to use monoclonal antibodies that target Il-1beta or the alpha receptor for IL-6 (IL-6Ralpha) — see below [49, 50].

The interaction of the ECs with the activated inflammatory cells and the role of the RANK-RANKL-OPG signalling axis in the formation of atherosclerotic lesions are further emphasised by the fact that both cell types express RANKL. RANKL produces its biological effects in the cells of the vascular wall by combining with RANK in the plasma membrane of these cells. Many of these effects on the ECs have been described above. Additionally, within VSMCs, RANKL, by combining with RANK, increases the activity of matrix metalloproteinases (MMPs). On the other hand, OPG, by binding to RANKL, prevents its association with RANK and inhibits the above-described influence of RANKL on the activity of MMPs in VSMCs [4, 48].

The increase in RANKL expression in ECs and VSMCs under the influence of oxidative stress has also been confirmed. This was confirmed for OxLDL and hydrogen peroxide (H_2_O_2_) [51]. The stimulating effect of the incubation of vascular wall cells with OxLDL on RANKL expression in these cells is particularly interesting, and this may reflect changes in the vascular wall of patients with high OxLDL concentrations. Within the vascular wall, RANKL has the ability to activate the degranulation of granulocytes infiltrating the vascular wall [52–54]. A protective effect in this respect is induced by FGF21, which reduces the expression of RANKL in the cells of the vascular wall. FGF21 is believed to play a protective role against endothelial damage, which is induced by oxidative stress, and plaque formation, as well as to protect against ischaemic damage to cardiomyocytes [55, 56].

The action of oxidative stress on vascular wall cells is related to the ubiquitin–proteasome system (UPS), which also plays a pathogenetic role in the development of endothelial dysfunction and atherosclerosis. It has also been confirmed that UPS plays an important role in the pathogenesis of congestive HF [57, 58]. In various experimental models of HF, both ischaemic and non-ischaemic, an increase in OPG expression was confirmed. Activation of the RANK-RANKL-OPG axis has been confirmed in a rat post-infarction HF model, and it is believed to be involved in the pathogenesis of HF [59].

### The combination of OPG and TRAIL

Many of OPG’s other mechanisms of action in the CV system may be due to its ability to bind to a ligand other than RANKL, such as TRAIL. TRAIL is a protein belonging to the TNF superfamily and is designated TNFSF 10. TRAIL expression has been confirmed in a wide variety of tissues, including vascular wall cells [3, 6, 60]. In humans, TRAIL is a type II transmembrane protein consisting of 281 amino acids. TRAIL, however, can be cleaved at the stalk domain, and after that, it can bind to two other TRAIL molecules to form a biologically active homotrimer circulating in the blood. TRAIL exerts its biological effect through a binding with its receptors. There are five types of receptors for TRAIL (TRAIL-R). TRAIL-R1 and TRAIL-R2 are agonist receptors and are known as death receptors (DRs). They are type I transmembrane proteins and contain an intracellular death domain (DD) which normally stimulates apoptosis when TRAIL connects to TRAIL-R1 and TRAIL-R2. The remaining receptors for TRAIL that are antagonist receptors are called decoy receptors (DcRs). These include TRAIL-R3, TRAIL-R4 and OPG. TRAIL-R3 and TRAIL-R4 are transmembrane proteins which, however, differ from DR in that they do not have a fully developed intracellular DD. On the other hand OPG is a soluble receptor. When TRAIL is not bound to a DcR, e.g. OPG, then it can bind to TRAIL-R1 and TRAIL-R2 and start a signalling pathway leading to apoptosis. These receptors have a high affinity for TRAIL, which in the form of a homotrimer binds to these receptors, and this results in trimerisation of these receptors. This in turn leads to the formation of a death-inducing signalling complex (DISC), which results in the accumulation of the adapter protein Fas-associated death domain (FADD). FADD is an intermediate link between DR and the pro-domain of the initiator caspase 8. At a later stage within the DISC, dimerisation of caspase 8 molecules occurs, leading to the formation of a mature caspase 8, which activates individual executive caspases, such as caspases 3, 6 and 7. This leads to cell apoptosis [6, 61, 62]. This is the so-called extrinsic apoptosis pathway. In some cell types, this activation of executive caspases must be additionally enhanced by the activation of an additional, internal, mitochondrial apoptotic pathway [6, 62]. In both cases, however, activation of executor kinases is necessary to induce apoptosis in cells sensitive to the pro-apoptotic effects of TRAIL. As for the two transmembrane DcRs for TRAIL (TRAIL-R3 and TRAIL-R4), they differ slightly from one another. TRAIL-R3 is linked to the plasma membrane with the help of a glycosylphosphatidylinositol (GPI) linker and lacks a cytoplasmic fragment. On the other hand, TRAIL-R4 has a shortened DD. Neither of them, however, can send a pro-apoptotic signal or start the apoptotic pathway when linked to TRAIL. Both of these DcRs have the ability to compete with DRs for binding to TRAIL, which, according to some researchers, may constitute a protective mechanism against the pro-apoptotic actions of TRAIL in cells resistant to these TRAIL actions [6]. Such a pro-apoptotic effect of TRAIL is shown in relation to neoplastically transformed cells, cells infected by a virus or the overstimulated cells of the immune system [3, 6, 62]. In studies on TRAIL-knockout mice, it was shown that the absence of TRAIL in these mice was associated with a greater susceptibility to the formation of metastases. This confirms the role of TRAIL in providing the body with protection against the formation and spread of neoplasms [3]. Some researchers have highlighted the fact that the selective induction of apoptosis by TRAIL takes place in transformed, neoplastic or infected cells but not in normal cells. The effect of TRAIL in normal cells, though, is less understood. It has been confirmed, however, that when TRAIL is combined with TRAIL-R1 and TRAIL-R2, the apoptotic pathway is not always triggered. Sometimes pathways responsible for cell survival are activated, such as ERK1/2 or PI3-kinase Akt [3, 62, 63]. It is believed that such differentiated effects of TRAIL in relation to different cells may be caused by the redistribution of individual types of TRAIL receptors or the inhibition of intracellular apoptotic pathways [3]. However, the results of the studies conducted so far are not clear-cut. Despite detailed studies of the relative proportions of individual types of receptors for TRAIL (DR/DcR) in individual types of cells, it is still not possible to fully predict the cell’s response to TRAIL [6, 64, 65]. Some differences in the effects of transmembrane TRAIL and soluble TRAIL on TRAIL-R1 and TRAIL-R2, however, can be highlighted. Transmembrane TRAIL has been shown to stimulate both types of receptors to the same extent. On the other hand, soluble TRAIL stimulates TRAIL-R1 to a greater extent, while it is less active on TRAIL-R2 [6, 66]. It is this TRAIL-R2 that is present to a greater degree on normal cells and which also explains in part their greater resistance to the pro-apoptotic effects of TRAIL. Most researchers, however, believe that the activation by TRAIL of the pathways either activating apoptosis or protecting against apoptosis depends mainly on the type of cell. Cells resistant to the pro-apoptotic effects of TRAIL include, but are not limited to, ECs and VSMCs, although both cell types possess TRAIL-R1 and TRAIL-R2 [6, 63, 67, 68]. Studies with human and rat VSMCs have shown resistance to the pro-apoptotic effects of TRAIL, even when TRAIL has been used at high concentrations (2 µg/ml) and when TRAIL incubation was extended to 48 h. For comparison, in the same experiment, TRAIL had induced apoptosis in HL-60 cells even at concentrations of 0.1 µg/ml [63]. It has also been shown that incubation of VSMCs with TRAIL prevents apoptosis induced by cytokines such as interferon gamma (IFN-gamma), TNF-alpha and IL-1beta. This study confirmed that TRAIL acts in VSMCs’ pro-survival, and also enhances migration and proliferation of these cells [63]. Similarly, pro-survival effects of TRAIL have been confirmed in studies on ECs. There are, however, a few differences in how TRAIL acts on ECs and VSMCs. Firstly, the ECs express all types of TRAIL receptors, and the VSMCs lack TRAIL-R3 and TRAIL-R4. Secondly, TRAIL activates the PI-3 kinase/Akt pathway in ECs more strongly than in VSMCs. Thirdly, blockade of the ERK pathway by its specific inhibitor PD98059 does not influence the protective, pro-survival effect of TRAIL in ECs, but, conversely, eliminates it in VSMCs [63]. This demonstrates the importance of the intracellular mechanisms that render the cell resistant to the pro-apoptotic effects of TRAIL. Activation of the ERKK 1/2 pathway is of particular importance in VSMCs [63]. It has been shown that TRAIL can simultaneously activate both the apoptotic pathway via DISC together with various pro-survival pathways such as PI-3 kinase/Akt/NO and proliferative pathways such as mitogen-activated protein kinase (MAPK)/ERK1/2 in various cell types [6, 65, 67–70]. It has also been confirmed in a mouse model of diabetic cardiomyopathy that systemic administration of TRAIL reduces cardiac fibrosis and cardiomyocyte apoptosis [71].

In contrast to OPG, TRAIL is present in significantly lower plasma concentrations in people with CAD than in healthy people [6, 34, 72–75]. It is the low plasma concentrations of TRAIL in this group of patients that are associated with a higher risk of CV mortality, a higher risk of post-infarction left ventricular remodelling and the development of HF [6, 34, 73, 76–78]. However, in patients with CAD, it is not only the plasma levels of TRAIL that are relevant. It was confirmed that in a group of patients with CAD, monocytes show significantly lower TRAIL expression than those monocytes of healthy people [74]. The low level of mRNA for TRAIL in monocytes in CAD patients correlated with the low plasma concentration of TRAIL in this group of patients. Monocytes/macrophages are believed to be a significant source of TRAIL in healthy subjects, and a significant reduction in the production of TRAIL by monocytes/macrophages in CAD may have pathogenetic implications related to atherosclerosis. Moreover, it was confirmed that interleukin-18 (IL-18) significantly reduces the expression of TRAIL in monocytes/macrophages. This is of particular importance because significantly elevated plasma concentrations of Il-18 have been confirmed in patients with CAD, and IL-18 itself has the ability to modulate the activity of various transcription factors, such as, inter alia, NF-kB [74]. In the group of patients with CKD, it was confirmed that low plasma concentrations of TRAIL are also an indicator of the progression of atherosclerotic lesions, as has been demonstrated in the carotid and femoral arteries [79]. Significantly lower TRAIL plasma concentrations were also confirmed in a group of patients with newly diagnosed type 2 diabetes compared to a control group without diabetes [73]. The introduction of lifestyle modifications and the introduction of pharmacological treatment for diabetes caused a significant increase in the plasma concentration of TRAIL in this group of patients; however, this concentration was still lower than in the group of healthy people. This study further confirmed that low TRAIL plasma concentrations in a pre-treatment group of type 2 diabetic patients were also associated with poorer endothelial function assessed in vivo on the brachial artery by FMD. An FMD reassessment of these patients 6 months after the start of diabetes treatment showed a significant improvement in endothelial function, but the results were still worse than in the non-diabetic control group [73]. TRAIL is considered a molecule with two faces. On the one hand, it is capable of inducing cell apoptosis and increasing inflammation; on the other hand, it protects cells by increasing their viability and inhibits inflammation. The mechanism of this differential activity of TRAIL is not fully understood [62]. Its concentration in different tissues or the presence of one type and the lack of other types of receptors for TRAIL may be of importance. In the case of ECs, however, all types of receptors have been found present in these cells, and they remain resistant to the pro-apoptotic effects of TRAIL. It is not known why ECs, under the influence of TRAIL, do not enter the apoptotic pathway, although they have all types of TRAIL receptors, including TRAIL-R1 and TRAIL-R2, which normally redirect the cell to the apoptotic pathway and thus kill it. One of the hypotheses is that TRAIL promotes apoptosis only in abnormal cells which have been changed, for example, by neoplastic transformation, but not in normal cells [3]. This mechanism mentioned above has been confirmed in ECs, where, despite the association of TRAIL with its receptors TRAIL-R1 and TRAIL-R2, apoptosis is not triggered, while pathways responsible for cell survival such as ERK1/2, PI3-Akt kinase are activated [3, 34, 62, 63]. The role of the individual pathways in modulating the cell’s response to TRAIL is complex. This applies in particular to the activation, in some cell types, of NF-kB, which exhibits broad pleiotropic effects. This makes it difficult to assess its role in modulating the response of individual cells to TRAIL [6, 80]. Various studies confirm TRAIL’s protective effect on blood vessels and its anti-atherosclerotic action. In a study using diabetic apolipoprotein E (ApoE)–knockout mice, which were given recombinant soluble TRAIL intraperitoneally, there was a significant reduction in the amount of atherosclerotic plaque formation [81]. In other studies in mice with double gene knockouts for ApoE and TRAIL, it was shown that the absence of TRAIL worsened the process of atherosclerotic plaque formation and its subsequent progression [82, 83]. Moreover, these studies found that TRAIL deficiency significantly contributed to plaque instability by increasing necrotic core and macrophage infiltration and by reducing VSMC and collagen content in atherosclerotic lesions [83]. Another study using mice with double knockouts of the TRAIL and ApoE genes showed that the lack of TRAIL resulted in a significant increase in the generation of ROS in the aortic wall of the mice tested [72]. Increased vascular ROS production is a recognised factor in causing endothelial dysfunction and promoting the development of atherosclerotic lesions. The same study confirmed that TRAIL-deficient mice exhibited a significant impairment of the endothelial-dependent aortic relaxation response to acetylcholine, while the endothelial-independent relaxation response to sodium nitroprusside was preserved [72]. In vitro studies with cultured ECs confirmed that Ang II, by activating the AT1 receptor, increases the production of ROS [72]. The same study confirmed that prior incubation of ECs with low concentrations (1 ng/ml) of TRAIL prior to incubation with Ang II significantly inhibited ROS production by ECs when compared with incubating these cells with Ang II alone. This confirms that TRAIL inhibits Ang II–induced ROS production in ECs and protects against endothelial dysfunction [72]. This was confirmed in vitro in cultured human microvascular endothelial cells (HMECs) when these cells were incubated with low concentrations (1 ng/ml) of TRAIL. In this study, TRAIL was shown to inhibit Ang II–induced ROS production in the ECs mainly by inhibiting reduced nicotinamide adenine dinucleotide phosphate (NADPH) oxidase 4 (NOX-4) [72]. However, the molecular mechanisms behind this TRAIL action are not fully understood. It is believed that the regulation of increased ROS production in ECs under the influence of Ang II is complex and requires the interaction of various enzymes, including NOX-4 and uncoupled eNOS, and the involvement of mitochondria [72, 84, 85]. The finding that TRAIL inhibits Ang II–induced ROS production in HMEC by inhibiting NOX-4 activity is in contrast to previous studies by the same authors who showed that TRAIL increased NOX-4 expression and increased H_2_O_2_ production in ECs. However, as the authors themselves stress, this may be due to the different conditions under which the ECs were tested in these studies. In the earlier studies, HMEC was tested in vitro under baseline conditions without Ang II exposure [62, 86]. Moreover, TRAIL was used in much higher concentrations, i.e. in the range of 10–400 ng/ml. As shown in this study, at this concentration range, TRAIL stimulated proliferation, migration and the tubule formation of ECs, in part by increasing NOX-4 expression and enhancing H_2_O_2_ production. At a certain concentration range, H_2_O_2_, produced with the participation of NOX-4, can stimulate the proliferation and migration of ECs. It has been further confirmed that under certain conditions NOX-4 can stimulate eNOS phosphorylation at Ser^1177^ and enhance NO production. These studies have confirmed that TRAIL regulates the endothelial function in the area of angiogenesis in vitro, through, inter alia, the modulation of H_2_O_2_ production, eNOS phosphorylation and NO production, and does so by modulating NOX-4 expression and activity [62, 86]. Thus, the effect of TRAIL on the expression and activity of NOX-4 in ECs may vary depending on whether basal conditions or pathology, including the effect of high levels of Ang II on the ECs, is involved.

It has also been confirmed that TRAIL inhibits calcification of blood vessels. In a study using TRAIL-deficient mice, a significant increase in RANKL expression was observed, which led to over-calcification of the vascular wall in these mice [87]. This protective effect of TRAIL against calcification has also been confirmed in vitro [87, 88]. In vitro studies, in which both ECs and VSMCs were assessed, showed that TRAIL significantly weakened RANKL-mediated osteoblastic signals in these cells [88]. In vitro, TRAIL also protected VSMCs exposed to calcium ions from calcification [87].

The exact molecular mechanisms of the anti-atherosclerotic effects of TRAIL are, so far, poorly understood. One of the concepts is that TRAIL has an anti-atherosclerotic effect, through its protection of normal endothelial cells and its strong anti-inflammatory action. This happens mainly by activating the above-mentioned pathways, such as ERK1/2 or PI3-kinase Akt, which are known for promoting cell survival [3, 89]. In vitro studies have shown that TRAIL significantly increases the activity of eNOS by its phosphorylation at Ser^1177^ and increases NO production in ECs [90–93]. TRAIL, however, does not activate NF-kB in the ECs. The augmented phosphorylation of eNOS and the increase in NO production in ECs under the influence of TRAIL occur through the activation of the PI3 kinase/Akt pathway (Fig. 2). This study confirmed that the use of a specific pharmacological inhibitor of the PI3 kinase/Akt pathway, LY294002, completely abolished TRAIL-induced NO production in ECs [68]. Additionally, TRAIL has been shown to induce prostaglandin E2 (PGE2) production in ECs, mainly by increasing COX-1 activity, as confirmed in vitro in endothelium cell culture conditions using a specific COX-1 inhibitor, SC-560 [3]. Particularly noteworthy is the strong stimulation of eNOS activity by TRAIL and the increase of NO production in ECs, due to the key and multidirectional role of NO in maintaining homeostasis in the CV system. NO released from ECs plays a key role in regulating the flow in the vascular system. In addition, NO is a regulator of a wide variety of processes such as platelet and leucocyte adherence to endothelial surfaces, platelet aggregation, VSMC proliferation and angiogenesis. An important difference between TRAIL and other compounds which also actively stimulate the release of NO from ECs, such as bradykinin, is that all these other compounds simultaneously activate NF-kB, while TRAIL increases eNOS activity and NO production without simultaneously activating NF-kB [67]. This is of particular importance due to the well-known fact that NO exerts its anti-inflammatory and anti-atherosclerotic effects mainly by inhibiting the activity of NF-kB. It is also known that in the early stages of development of endothelial dysfunction, activation of NF-kB occurs, which triggers inflammation, adherence of leucocytes to the endothelium and the progression of atherosclerosis. The fact that TRAIL does not activate NF-kB means that it does not increase the expression of ICAM-1, VCAM-1 and E-selectin in ECs and prevents increased adherence of leucocytes to the endothelial surface [6, 67]. In addition, TRAIL inhibits the pro-adhesion action of cytokines such as TNF-alpha and IL-1beta through a specific inhibitory effect on chemokines such as chemokine C–C motif ligand 8 (CCL8)/monocyte chemoattractant 2 protein (MCP-2) and C-X-C motif chemokine ligand 10 (CXCL10)/interferon gamma-induced protein 10 (IP-10) [94]. NO, whose production by eNOS is stimulated by TRAIL, is an endogenous inhibitor of NF-kB and COX-2 activity and, at the same time, increases the activity of COX-1. The effect of TRAIL in stimulating COX-1 activity and PGE2 production is believed to be accomplished by increasing NO release (Fig. 2). This was confirmed by the finding that the addition of the eNOS inhibitor L-NAME to the EC culture inhibited TRAIL-induced PGE2 secretion [68]. Another study showed that TRAIL has the ability to induce NO release in VSMCs as well. This was confirmed in in vitro studies in which rat VSMCs were incubated with recombinant TRAIL. It was found that TRAIL causes a significant, dose-dependent increase in NOS activity and an increase in intracellular cGMP concentration in VSMCs [95]. The same study confirmed that insulin causes downregulation of TRAIL expression in rat and human VSMCs. This is consistent with the observation that diabetic patients treated with insulin have significantly lower plasma TRAIL concentrations in comparison with either diabetic patients not treated with insulin or those without diabetes. The same was confirmed in rats with streptozotocin-induced diabetes, treated and not treated with insulin. The molecular mechanism of this effect of insulin on TRAIL expression is unknown. However, the potential pathophysiological significance of this phenomenon is under discussion [95]. In an experimental model of rats with diabetes mellitus induced with intraperitoneal injections of streptozotocin (STZ), the effect of the intraperitoneal administration of recombinant TRAIL (rTRAIL) on the glycemic parameters was also investigated [93]. It has been shown that the intraperitoneal administration of rTRAIL resulted in a significant reduction in fasting glucose and HBA1C compared to the placebo group. The same study also evaluated the endothelial function in vitro based on the assessment of endothelial-dependent, acetylcholine-induced relaxation [93]. Aortic rings from healthy rats were incubated under normoglycaemia and hyperglycaemia conditions in the presence and absence of rTRAIL. Additionally, a specific PI3 kinase/Akt inhibitor, i.e. LY294002, and the eNOS inhibitor, i.e. L-NAME, were used for this incubation. Aortic rings incubated at high glucose concentrations showed a significantly impaired relaxation response to acetylcholine compared to the control rings. In this experiment, TRAIL was shown to significantly improve the endothelial-mediated relaxation response to acetylcholine in aortic rings incubated under hyperglycaemic conditions. Moreover, it was confirmed that this effect is reversed by adding LY294002 and L-NAME to the incubation; i.e. it is dependent on the PI3 kinase/Akt pathway and eNOS activity [93].

All these activities of TRAIL, demonstrated in recent studies, especially its ability to increase eNOS activity and NO production, indicate an important vasoprotective role of TRAIL in the vascular system. At the same time, all of these TRAIL actions can potentially be antagonised by OPG, which is a decoy receptor capable of binding to TRAIL, which blocks its binding to the receptors present in the ECs and VSMCs. The proportions of OPG and TRAIL concentrations in the local vascular environment in specific clinical situations may prove crucial.

## Clinical importance and future perspectives

The mechanisms of action of OPG and the RANK-RANKL-OPG and OPG-TRAIL signalling axes are complex and still not fully understood. Their participation in the pathogenesis of CVDs is of continued interest, and the RANK-RANKL-OPG-TRAIL axis itself is seen as a potential therapeutic target in CVDs. Several areas are of particular interest.

One of these is the interaction of OPG with TRAIL and the inhibition of TRAIL-induced MSC migratory activity. Of great interest is the inhibitory influence of OPG on the stimulating effect of TRAIL in relation to the migratory activity of MSCs which has been demonstrated in vitro. This is due, inter alia, to the confirmed, increased mobilisation of MSCs from the bone marrow during the acute phase of MI and their migration to the infarcted area of the myocardium. The participation of these cells in the regeneration of the heart muscle and protection against unfavourable post-infarction remodelling of the left ventricle is under consideration [23]. This is an attractive concept in the context of the above-presented relationship between the amount of MSCs in the peripheral blood and the development of post-infarction HF. TNF-alpha is also strongly involved in these mechanisms, and appears to influence the MSC mobilisation process in various ways. On the one hand, it strongly increases the MSC response to TRAIL and thus contributes to a greater mobilisation of MSCs from the bone marrow into the peripheral blood. On the other hand, it also strongly stimulates the production of OPG by ECs and VSMCs in the acute phase of MI, and OPG strongly inhibits the activity of TRAIL in relation to MSC. High concentrations of OPG have been shown to inhibit the stimulating effect of TRAIL on the migratory capacity of MSCs. In vitro studies revealed that the neutralisation of OPG by specific antibodies restored TRAIL activity, stimulating the migration capacity of MSCs. As described above, clinical trials confirm that high concentrations of OPG, and especially a high OPG/TRAIL ratio, in the acute phase of MI are associated with a significantly higher risk of developing both adverse post-infarction left ventricular remodelling and HF, as well as a higher risk of death. The mechanisms by which OPG, TRAIL and MSC interact and by which this process is regulated are complex and only partially understood. Due to the demonstrated activity of TRAIL in relation to MSC and its anti-inflammatory and anti-atherosclerotic effects, however, the regulation of TRAIL activity by TNF-alpha and OPG is of continuing interest. Perhaps the TNF-alpha-dependent increase in OPG concentrations will prove to have significant pathogenetic implications for acute MI by the neutralisation of the beneficial effects of TRAIL on MSCs and on the vascular system. In this context, the ability to disable the action of OPG is an attractive potential therapeutic concept in CVDs.

Ways of neutralising the inhibitory effects of OPG on TRAIL, where it acts upon the vascular wall cells, are also being sought. The effects of TRAIL, confirmed in studies, of stimulating eNOS and increasing the production of NO in ECs, are of particular interest. More research is needed to determine whether intervention in the range of TRAIL and OPG concentrations and their ratios will yield clinical benefits in terms of CVDs. The studies conducted so far serve as encouragement in the search for ways to promote the effects of TRAIL in the vascular system while, at the same time, eliminating the negative effects of OPG. It is not known whether ways to do this can be found and whether their implementation will achieve clinical benefits. However, the known mechanisms of action of both OPG and TRAIL in the CV system undoubtedly make this OPG/TRAIL system an attractive potential therapeutic target in CVDs.

UPS may also be of potential importance in the therapy of HF due to its role in the occurrence of endothelial dysfunction and atherosclerosis induced by oxidative stress, as well as in the development of HF [57, 58]. Similarly, individual elements of the RANK-RANKL-OPG signalling axis are associated with the development of ischaemic and non-ischaemic HF, which has been confirmed in various experimental HF models [59]. However, the detailed molecular mechanisms of the action of the RANKL-RANK-OPG axis in the development of HF remain unclear and require further studies.

Additionally, the interaction of OPG with various pro-inflammatory cytokines and the ability of OPG to increase the expression of adhesion molecules in ECs became the basis for the search for new therapeutic strategies in CVDs. OPG is considered to be a chemotactic factor for inflammatory cells infiltrating the vascular wall at an early stage of the development of atherosclerotic lesions [4, 17, 44–48]. Activated inflammatory cells are a source of pro-inflammatory cytokines such as IL-6 and IL-1beta, among others. IL-6 exerts its pro-inflammatory effects mainly by binding to its soluble IL-6Ralpha receptor. It is these cytokines and their receptors that are considered to be potential therapeutic targets in CVDs. Canakinumab is a human monoclonal antibody directed against Il-1beta that has been approved in both the USA and Europe for the treatment of chronic immune-related inflammatory diseases such as cryopyrin-associated periodic syndrome (CAPS), systemic juvenile idiopathic arthritis (SIJA) and gout inflammation in the joints. Other human monoclonal antibodies such as sarilumab and tocilizumab have also been approved by the US Food and Drug Administration (FDA) and the European Medicines Agency (EMA) for the treatment of rheumatoid arthritis. Sarilumab and tocilizumab are human monoclonal antibodies directed against the IL-6Ralpha receptor. It is IL-1beta and IL-6, produced in large amounts by activated inflammatory cells, that induce and sustain inflammation within the vascular wall in the early stages of atherosclerotic lesions. Therefore, sarilumab and tocilizumab are currently seen as potential CVD treatment that could also be used in these cardiovascular indications in the future.

## Conclusions

Much has changed in the understanding of the importance of OPG in the CV system since the first observations made in OPG knockout mice. It was then noted that, in addition to increased osteoporosis and a greater frequency of bone fractures, these mice also had increased calcification of the aortic wall and renal arteries [6, 96–98]. These first observations became the basis for the perception of OPG as a factor protecting vessels against calcification. The similarities between the regulation of osteoclastogenesis in bone and the calcification of the vascular wall in addition to the contribution to this regulation of the RANK-RANKL-OPG signalling axis seemed to support this hypothesis. Later studies, however, showed that older women with severe osteoporosis have high concentrations of OPG, and the higher the levels of OPG, the more advanced their osteoporosis. These high concentrations of OPG contrasted with women of the same age without osteoporosis [6, 99, 100]. Moreover, high concentrations of OPG in these women strongly and positively correlated with CV mortality [6, 101, 102]. Further studies provided evidence that there is a strong, positive correlation between OPG concentration and the presence and severity of CAD [3, 6, 103–105]. It has also been confirmed that the increase in OPG concentration in the course of acute coronary syndromes is an indicator of the risk of adverse CV events and poor prognosis [20, 21]. This was initially considered to be a protective mechanism, in the belief that the concentration of OPG increases in response to the action of vascular disrupting agents, and that high levels of OPG are intended to be protective or at least a marker of the disrupting power of other factors. Later studies, however, provided substantial evidence of a direct, unfavourable effect of OPG on the blood vessel wall. The mechanisms of OPG action on ECs and VSMCs which are so far known are given above. The unfavourable effects of OPG on elements of the vascular wall include, among others, enhancement of the adherence of leucocytes to the endothelial surface, the activation of RAS, pro-inflammatory and pro-fibrotic effects and the induction of endothelial dysfunction in the early stages of atherogenesis. The known mechanisms of the action of OPG on vascular wall cells now allow us to better understand the clinical observations indicating a strong relationship between high OPG concentrations and the presence and course of CVDs, as well as the development of HF and CV mortality. It is currently believed that, although initially the increase in OPG concentration may be a reaction of the vascular wall to various disrupting factors, in the later stages, persistence of high OPG concentrations triggers the above-mentioned OPG unfavourable mechanisms of action, which ultimately contribute to vascular damage and atherogenesis.

These complex mechanisms of action of OPG in the cells of the vascular wall are currently considered the most important for the pathogenesis of CVDs and potential future CVD treatments. Researchers are particularly interested in the mutual interactions of OPG with its ligands, RANKL and TRAIL. In terms of potential clinical benefits, TRAIL looks the most promising. It has many beneficial anti-atherosclerotic effects and has been shown to neutralise the adverse effects of OPG on the vascular wall. Research is underway on how best to neutralise the inhibitory effects of OPG on TRAIL, especially its effects on cells in the CV system. The stimulation of eNOS and the increase in the production of NO in ECs, which have been confirmed as effects of TRAIL, are particularly interesting. The OPG and TRAIL concentration ratio in the local vascular environment seems to be very important. The mechanisms of the OPG/TRAIL interaction and the mechanisms of their action on the cells of the vascular wall are complex and still not fully understood. Despite this, ways are being sought to eliminate the unfavourable OPG effects while, at the same time, promoting the effects of TRAIL in the vascular system. Further research is required to investigate whether ways can be found to do this and, if so, whether they will provide clinical benefits. Without doubt, however, the OPG and TRAIL mechanisms of action in the CV system mean that this signal axis is a potentially attractive therapeutic target in CVDs.
